# Prevalence of iron deficiency in patients with mild to moderate bleeding disorders and bleeding disorder of unknown cause

**DOI:** 10.1016/j.rpth.2025.102999

**Published:** 2025-08-11

**Authors:** Tim Dreier, Dino Mehic, Justin Oosterlee, Jasmin Rast, Alexandra Kaider, Helmuth Haslacher, Cihan Ay, Ingrid Pabinger, Johanna Gebhart

**Affiliations:** 1 Division of Hematology and Hemostaseology, Department of Medicine 1, Medical University of Vienna, Vienna, Austria; 2 Center for Medical Data Science, Institute of Clinical Biometrics, Medical University of Vienna, Vienna, Austria; 3 Department of Laboratory Medicine, Vienna, Austria

**Keywords:** anemia, iron-deficiency, blood coagulation disorders, von Willebrand diseases, ferritin

## Abstract

**Background:**

Iron deficiency (ID) and ID anemia (IDA) are often caused by chronic bleeding, especially heavy menstrual bleeding, and thus may occur at a high frequency in patients with mild to moderate bleeding disorders (MBDs).

**Objectives:**

To study the prevalence of iron deficiency in mild to moderate bleeding disorders and bleeding disorder of unknown cause.

**Methods:**

The iron status of patients with MBD from the Vienna Bleeding Biobank, a prospective cohort study, was analyzed and compared with age- and sex-matched healthy controls. ID was defined as ferritin ≤30 μg/L, transferrin saturation <16%, or iron therapy at inclusion. IDA was defined as hemoglobin <12 g/dL in women and <13 g/dL in men diagnosed with ID.

**Results:**

The rates of ID and IDA were comparable between 646 patients with MBD and 118 controls, as 250 patients with MBD (39%) had ID and 40 (6%) had IDA, compared with 37 controls with ID (31%) and 6 with IDA (5%). von Willebrand disease showed a significantly higher rate of ID than controls (49%) before correction for multiple testing, while there was no significant difference between other MBD diagnoses and controls (bleeding disorder of unknown cause: 39%; platelet function disorders: 33%; and coagulation factor deficiencies: 28%). In multivariable regression, we identified female sex, younger age, and higher body mass index, but not MBD diagnoses, bleeding score, or blood group O, associated with ID.

**Conclusion:**

ID was common among MBDs, especially von Willebrand disease and female patients, but also in controls. Our data highlight the importance of assessing iron status in patients with MBDs, especially in young female individuals, regardless of the presence of bleeding symptoms.

## Essentials

• Iron deficiency is a common comorbidity in patients with bleeding disorders.
• Iron deficiency and consequent anemia were assessed in patients with bleeding disorders.
• More than one-third of patients and controls were iron-deficient, but anemia was rare.
• Our results highlight the importance of iron-status testing, regardless of bleeding symptoms.

## Graphical abstract

Created in BioRender. T. Dreier (2025) https://BioRender.com/dikjwlr

**Figure:**
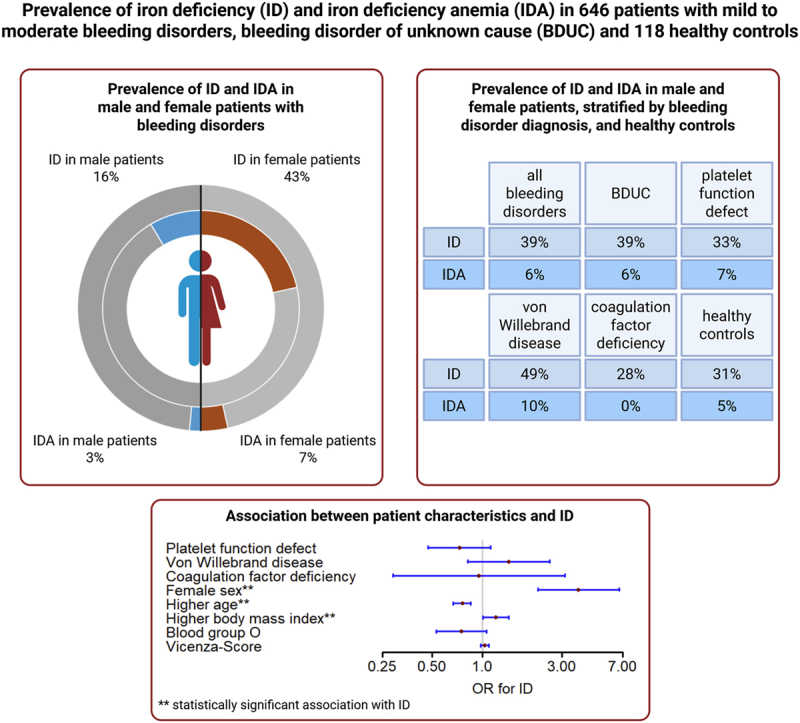

## 1 Introduction

Iron deficiency (ID) and ID anemia (IDA) are common complications in patients with chronic bleeding and, therefore, may be a relevant issue in patients with inherited or acquired bleeding disorders [1,2]. In these patients, chronic blood loss can lead to an increased demand for iron that exceeds the dietary intake, eventually depleting iron stores and impairing hemoglobin biosynthesis [3]. ID and IDA can affect overall quality of life [4] and are associated with fatigue, weakness, mood swings, and impaired cognitive function [3,5]. Overall, ID and IDA impose a high burden on affected individuals [6].

Mild to moderate bleeding disorders (MBDs) are inherited conditions that result in an increased bleeding tendency, manifested by lifelong bleeding symptoms such as easy bruising and epistaxis [[7], [8], [9]]. However, patients with MBDs are also at an increased risk of severe and potentially life-threatening bleeding complications following hemostatic challenges, such as postoperative or postpartum bleeding [10]. Established diagnoses of MBDs include platelet function disorders, von Willebrand disease, and coagulation factor deficiencies [9,11]. However, the majority of patients with MBDs are currently diagnosed with bleeding disorder of unknown cause (BDUC), reflecting normal results on a comprehensive hemostatic evaluation [12]. Patients with BDUC present with bleeding symptoms and severity similar to those with an established diagnosis of MBD [13].

Heavy menstrual bleeding is most commonly associated with ID or IDA and is considered a hallmark of women and persons assigned female at birth with inherited bleeding disorders [1,5,14,15]. However, heavy menstrual bleeding is also highly prevalent in the otherwise healthy adult female population [16]. To date, data on the prevalence of ID and IDA in patients with MBDs, especially BDUC, are scarce [17].

Since 2009, the Vienna Bleeding Biobank has been investigating adult patients with MBDs, including a majority of patients with BDUC, with the aim of characterizing these patients and elucidating the underlying pathomechanisms and factors associated with the bleeding phenotype in MBDs. Importantly, we have previously shown an overrepresentation of female patients in our MBD patient cohort, potentially identifying a group of patients at particularly high risk of ID and IDA [18].

The aim of this study was to investigate the incidence of ID and IDA in our large and well-characterized cohort of patients with MBD and BDUC from the Vienna Bleeding Biobank in comparison with age- and sex-matched healthy controls. We analyzed the association of iron status with patient clinical data and the bleeding phenotype. This study addresses the importance of iron-status testing in MBD patients and may identify subgroups at a particularly high risk of ID or IDA.

## 2 Methods

### 2.1 Study design and patients

Data from patients in the Vienna Bleeding Biobank, a single-center prospective cohort study on adult patients with MBDs, were analyzed. The Vienna Bleeding Biobank is managed at the hemostaseology outpatient clinic of the Vienna General Hospital (Division of Hematology and Hemostaseology, Medical University of Vienna) and includes adult patients referred for the evaluation of a bleeding tendency. Inclusion and exclusion criteria for the Vienna Bleeding Biobank have been published previously [9] and are outlined in Supplementary Table S1. The study was approved by the Ethics Committee of the Medical University of Vienna (Ethics committee number 603/2009) in accordance with the Declaration of Helsinki of 1975 and its amendments/revisions. Age- and sex-matched healthy controls were selected from the Vienna Bleeding Study (Ethics committee number 039/2006).

Patients and healthy controls gave written informed consent before inclusion into either study. At inclusion, a structured interview regarding the patients’ or controls’ general medical and bleeding history was conducted by a trained professional using a standardized structured questionnaire. The patients’ and controls’ clinical bleeding phenotype was assessed using the Vicenza bleeding score, a standardized scoring system for MBDs evaluating 11 bleeding symptoms for their presence and severity [19]. The cutoffs for the Vicenza bleeding score are ≥3 for men and ≥5 for women of all ages. Details on the evaluation of bleeding symptoms using the Vicenza bleeding score are outlined in the Supplementary Methods.

After the interview, blood was drawn for analysis and storage in the biobank. Details on blood sampling and storage are summarized in the Supplementary Methods, and sample analyses by a panel of plasmatic coagulation and platelet function tests are summarized in Supplementary Table S2. Diagnostic criteria for MBD diagnoses have been applied as previously published and are depicted in Supplementary Figure S1 [20]. Measurement of iron status was conducted at study inclusion from fresh serum samples.

Until October 2023, 938 patients with MBD were included in the Vienna Bleeding Biobank. Data on iron status were available for 646 patients (69%), who were included in this analysis, and compared with data of 118 age- and sex-matched healthy controls. The remaining 292 patients were excluded from this study due to incomplete or missing iron-status data. Of these, 141 (15%) had partial iron-status data, with measurements of either ferritin or transferrin saturation, while the remaining 151 patients (16%) had no iron-status assessment. There were no differences in the proportion of female patients, median age, prevalence of blood group O, or median bleeding score between patients with a full iron status, a partial iron status, or no iron parameters on file, as outlined in Supplementary Table S3.

### 2.2 Diagnosis of ID and IDA

ID was diagnosed in individuals with ferritin levels of ≤ 30 μg/L and/or a transferrin saturation level of <16%. Furthermore, patients who were on iron supplementation at study inclusion were also classified as iron-deficient, regardless of their iron status at inclusion. In accordance with the World Health Organization guidelines, anemia was defined as hemoglobin levels of <12 g/dL in women and <13 g/dL in men [21]. IDA was diagnosed in individuals with ID and anemia.

Ferritin was measured using a nephelometry assay according to previously outlined standards [22]. Transferrin was measured using immunoturbidimetry, and transferrin saturation was calculated based on these measurements. All parameters of iron status were measured at the Department of Laboratory Medicine, Medical University of Vienna, Austria, with methods accredited according to ISO 15189:2012.

### 2.3 Statistical analysis

Continuous data are described by the mean (SD) or, in the case of nonnormally distributed values, by the median (IQR). Categorical variables are represented as counts (percentages). An unpaired Student’s *t*-test was used to compare continuous variables between patients with MBD and the healthy control group. Analysis of variance models were calculated for comparisons of the 5 groups (healthy controls/BDUC/platelet function defects/von Willebrand disease/coagulation factor deficiencies). Post hoc pairwise comparisons were performed by applying the Tukey–Kramer multiplicity adjustment. Due to the skewed distribution of data, log_2_-fold–transformed ferritin levels were used for all statistical calculations. Categorical variables were compared by applying the chi-squared test or Fisher’s exact test, as appropriate. Post hoc pairwise comparisons were adjusted for multiplicity using the Bonferroni–Holm method. Univariate and multivariable logistic regression models were performed to evaluate the association of relevant clinical factors, MBD diagnosis, and the Vicenza bleeding score with the prevalence of ID in patients with MBD. Unadjusted and adjusted odds ratios (ORs) with 95% CIs were calculated to quantify the strength of associations. Two-sided *P* values of <.05 were considered statistically significant. SAS 9.5 (SAS Institute Inc) was used for all statistical calculations.

## 3 Results

### 3.1 Patient characteristics

In total, data of 646 patients with MBD and 118 age- and sex-matched healthy controls were analyzed. Of the patients with MBD, 133 (21%) were diagnosed with a platelet function defect, 63 (10%) with von Willebrand disease, and 18 (3%) with coagulation factor deficiencies. The remaining 432 patients (67%) were classified as BDUC. Detailed characteristics of patients with MBD, overall and according to diagnosis, and healthy controls are shown in Table 1.

**Table 1:** Clinical and laboratory characteristics of mild to moderate bleeding disorder patients overall and according to diagnoses, and of healthy controls.

Cohort	MBD	BDUC	PFD	VWD	CFD	HC
Cohort size, *n* (%)	646 (100)	432 (67)	133 (21)	63 (10)	18 (2)	118 (100)
White ethnicity, *n* (%)	633 (99)	426 (99)	126 (95)	63 (100)	18 (100)	118 (100)
Women, *n* (%)	540 (84)	371 (86)	109 (82)	54 (86)	6 (33)	94 (80)
Blood group O^a^, *n* (%)	305 (48)	191 (45)	59 (45)	46 (73)	9 (50)	17 (40)
Age (y), median (IQR)	40 (29-53)	41 (30-54)	37 (27-53)	33 (25-44)	45 (25-57)	46 (31-52)
BMI (kg/m^2^), median (IQR)	23 (21-27)	23 (21-27)	23 (20-26)	23 (20-26)	25 (23-29)	23 (21-25)
Vicenza BS, median (IQR)	5 (4-8)	5 (4-8)	5 (3-8)	5 (4-8)	5 (4-9)	0 (0-0)
No. of bleeding manifestations, median (IQR)	6 (4-7)	5 (4-7)	6 (4-7)	6 (5-8)	7 (6-9)	0 (0-0)
Hemoglobin (g/dL), mean (SD)	13.6 (1.3)	13.6 (1.2)	13.7 (1.3)	13.5 (1.4)	13.7 (2.0)	13.7 (1.2)
MCV (fL), mean (SD)	87.4 (4.5)	87.5 (4.5)	87.6 (4.6)	86.4 (4.6)	87.3 (3.2)	87.2 (4.2)
MCH (pg), mean (SD)	29.4 (1.8)	29.5 (1.8)	29.4 (1.9)	29.1 (1.9)	29.9 (1.2)	29.7 (1.8)
Platelet count (G/L), mean (SD)	251 (65)	251 (59)	244 (61)	271 (98)	245 (85)	261 (47)
Ferritin (μg/L), median (IQR)	49.9 (27.1-106.1)	50.3 (27.2-106.1)	61.9 (31.3-117.0)	31.2 (21.3-53.1)	92.6 (36.6-123.8)	54.2 (28.7-101.1)
Transferrin saturation (%), mean (SD)	24.4 (11.4)	24.1 (10.8)	24.0 (10.6)	27.6 (16.7)	24.2 (8.6)	25.4 (10.9)

BDUC, bleeding disorder of unknown cause; BMI, body mass index; BS, Bleeding Score; CFD, coagulation factor deficiency; HC, healthy control; MBD, mild to moderate bleeding disorder; MCH, mean corpuscular hemoglobin; MCV, mean corpuscular volume; PFD, platelet function disorder; VWD, von Willebrand disease.

a Blood group analysis was available for 640 patients with MBD (99%) and 42 HC (36%).

The majority of patients were female (*n* = 540, 84%), and the median age was 40 years. On average, patients reported 6 unique bleeding manifestations (IQR, 4-7), with a median Vicenza bleeding score of 5 (IQR, 4-8). Blood group O was more prevalent in 305 (48%) patients compared with 17 of 42 healthy controls (40%). There was no relevant difference between patients with MBDs and healthy controls in body mass index, hemoglobin level, mean corpuscular volume, mean corpuscular hemoglobin, or platelet count (Table 1).

Patients with BDUC presented with a similar proportion of women (*n* = 371, 86%) as those with platelet function defects (*n* = 109, 82%) and von Willebrand disease (*n* = 54, 86%), while patients with coagulation factor deficiencies showed the lowest proportion of female patients, with 33% (*n* = 6). The median age of patients with BDUC (41 years) was similar to that of patients with coagulation factor deficiencies (45 years) but was higher than that of patients with platelet function defects (37 years) or von Willebrand disease (33 years). Furthermore, the median Vicenza bleeding score was 5 in all diagnostic subgroups, and thus similar in patients with BDUC compared with patients with established bleeding disorder diagnoses. However, patients with BDUC had the lowest number of bleeding manifestations (median, 5) compared with patients with platelet function defects (median, 6), von Willebrand disease (median, 6), and coagulation factor deficiencies (median, 7). Lastly, body mass index, hemoglobin level, mean corpuscular volume, mean corpuscular hemoglobin, and platelet count were similar between patients with BDUC and patients with established diagnoses of a bleeding disorder.

Importantly, blood group O was generally highly prevalent in patients with MBD (48%), among whom patients with von Willebrand disease showed the highest rate in 46 patients (73%), while there was no difference in the occurrence of blood group O among the other MBD diagnoses (Table 1).

### 3.2 Ferritin and transferrin saturation in patients with MBD and BDUC

The median ferritin level in patients with MBD was 49.9 μg/L (IQR, 27.1-106.1 μg/L), which was not significantly different from that of healthy controls, with 54.2 μg/L (IQR, 28.7-101.1 μg/L; *P* = .876). Furthermore, there was no difference in the mean (SD) transferrin saturation between patients with MBD (24.4% [11.4%]) and healthy controls (25.4% [10.9%]; *P* = .429; Table 1, Supplementary Figure S2).

Analysis of variance revealed statistically significant differences in ferritin levels when comparing all 5 groups (healthy controls/BDUC/platelet function defects/von Willebrand disease/coagulation factor deficiencies; *P* < .001). Patients with BDUC had a median ferritin level of 50.3 μg/L (IQR, 27.2-106.1 μg/L), which was similar to the ferritin levels of healthy controls (54.2 μg/L; IQR, 28.7-101.1 μg/L; Tukey–Kramer adjusted *P* value [*P*_adj_] = 1.0), as well as patients with platelet function defects (61.9 μg/L; IQR, 31.3-117.0 μg/L; *P*_adj_ = .525) and patients with coagulation factor deficiencies (92.6 μg/L; IQR, 36.6-123.8 μg/L; *P*_adj_ = .39). However, ferritin levels of patients with BDUC were significantly higher than those of patients with von Willebrand disease, who had the lowest ferritin levels (31.2 μg/L; IQR, 21.3-53.1 μg/L; *P*_adj_ = .006). Ferritin levels in patients with von Willebrand disease were also lower than those of healthy controls (*P*_adj_ = .035; Table 1, Supplementary Figure S2).

No statistically significant difference was detected in transferrin saturation levels across all 5 groups (overall *P* = .185). Patients with BDUC had similar levels of transferrin saturation of 24.1% (SD, 10.8%) as healthy controls (mean [SD], 25.4% [10.9%]; *P*_adj_ = .84), patients with von Willebrand disease (mean [SD], 27.6% [16.7%]; *P*_adj_ = .146), patients with platelet function defects (mean [SD], 24.0% [10.6%]; *P*_adj_ = 1.0), or patients with coagulation factor deficiencies (mean [SD], 24.2% [8.6%]; *P*_adj_ = 1.0).

### 3.3 Prevalence of ID and IDA in patients with MBD and BDUC

A total of 250 patients with MBD (39%) met the diagnostic criteria for ID (Table 2, Supplementary Figure S3). Of these patients, 113 (44%) had a ferritin level of ≤30 μg/L, 60 (24%) had a transferrin saturation of <16%, and 75 (30%) were below the cutoff for both parameters. In addition, 2 patients were classified as iron-deficient due to iron supplementation at study inclusion. However, rates of ID were similarly high in the control cohort, with 37 of 118 healthy controls (31%) being iron-deficient at study inclusion (*P* = .13). Of these, 30 (81%) had ferritin levels of ≤30 μg/L, 20 (54%) had a transferrin saturation of <16%, and 13 (35%) had low levels of both ferritin and transferrin saturation.

**Table 2:** Prevalence of iron deficiency and iron deficiency anemia in patients with mild to moderate bleeding disorder overall and according to mild to moderate bleeding disorder diagnoses, and for healthy controls.

Iron status in MBD	MBD	BDUC	PFD	VWD	CFD	HC
Total cohort	*n* = 646	*n* = 432	*n* = 133	*n* = 63	*n* = 18	*n* = 118
ID, *n* (%)	250 (38.7)	170 (39.4)	44 (33.1)	31 (49.2)	5 (27.8)	37 (31.4)
*P* value vs HC	.13	.11	.77	**.018**^a^	.76	
IDA, *n* (%)	40 (6.2)	25 (5.8)	9 (6.8)	6 (9.5)	0 (0)	6 (5.1)
*P* value vs HC	.64	.769	.575	.347	1.0	

Iron status in MBD	MBD	BDUC	PFD	VWD	CFD	HC
Women < 55 y	*n* = 428 (79%)	*n* = 286 (77%)	*n* = 85 (78%)	*n* = 28 (52%)	*n* = 6 (100%)	*n* = 72 (77%)
ID, *n* (%)	205 (48.0)	142 (49.7)	33 (38.8)	28 (54.9)	2 (33.3)	29 (40.3)
*P* value vs HC	.23	.155	.85	.109	1.0	
IDA, *n* (%)	31 (7.2)	20 (7.0)	6 (7.0)	5 (9.8)	0 (0)	6 (8.3)
*P* value vs HC	.74	.695	.765	.76	1.0	

The reported *P* values are the results of chi-squared tests and Fisher’s exact tests comparing the respective MBD diagnosis with healthy controls. The overall comparisons (HC/BDUC/PFD/VWD/CFD) using the chi-squared test were not statistically significant (total cohort: ID: *P* = .09; IDA: *P* = .579; women < 55 years: ID: *P* = .19; IDA: *P* = .897).

BDUC, bleeding disorder of unknown cause; CFD, coagulation factor deficiency; HC, healthy control; ID, iron deficiency; IDA, iron deficiency anemia; MBD, mild to moderate bleeding disorder; PFD, platelet function defect; VWD, von Willebrand disease.

a Significance did not prevail after correction for multiple testing using the Bonferroni–Holm method.

Patients with BDUC had an ID prevalence of 39% (170 patients), which was similar to that of healthy controls of 31% (*P* = .11). Similarly, we observed no statistically significant difference in the ID rate between patients with BDUC and patients with platelet function defects (33%; *P* = .19), von Willebrand disease (49%; *P* = .137), and coagulation factor deficiencies (28%; *P* = .32). Patients with von Willebrand disease showed the highest prevalence of ID, which was significantly higher than that of healthy controls (*P* = .018). However, this significance did not prevail after Bonferroni–Holm correction for multiple testing.

As outlined in Table 2, IDA occurred at a similar frequency in patients with MBD (*n* = 40, 6%) and healthy controls (*n* = 6, 5%; *P* = .64). The occurrence of IDA was similar between patients with BDUC (*n* = 25, 6%) and healthy controls (*P* = .769), patients with platelet function defects (*n* = 9, 7%; *P* = .678), and von Willebrand disease patients (*n* = 6, 10%; *P* = .26). None of the patients with coagulation factor deficiencies had IDA (*P* = .61 compared with BDUC). Patients with von Willebrand disease again showed the highest rate of IDA, albeit with a nonsignificant difference to healthy controls (*P* = .347).

### 3.4 Correlation of clinical parameters with ID in patients with MBD

We next analyzed the impact of clinical factors on the occurrence of ID in patients with MBD (Table 3). None of the MBD diagnoses were significantly associated with ID in univariate or multivariable logistic regression analysis. Female sex (univariate OR, 3.68; 95% CI, 2.16-6.29) and younger age (univariate OR, 0.79; 95% CI, 0.71-0.88) were significantly associated with ID, whereas body mass index, blood group O, and the Vicenza bleeding score were not associated with ID in univariate regression analysis. After adjustment for these clinical parameters, age and female sex remained significantly associated with ID in multivariable logistic regression analysis. In this model, body mass index also reached a barely significant association with ID (multivariable OR, 1.04; 95% CI, 1.00-1.08).

**Table 3:** Association of clinical parameters and iron deficiency in patients with mild to moderate bleeding disorders.

Parameters	Univariate regression, OR (95% CI)	Multivariable regression, OR (95% CI)
Total cohort		
MBD diagnosis (vs BDUC)		
PFD	0.76 (0.51-1.15)	0.73 (0.47-1.12)
VWD	1.49 (0.88-2.54)	1.44 (0.82-2.53)
CFD	0.59 (0.21-1.69)	0.95 (0.29-3.12)
Female sex	**3.68 (2.16-6.29)**	**3.78 (2.15-6.63)**
Age (unit = 10 y)	**0.79 (0.71-0.88)**	**0.76 (0.67-0.85)**
BMI (unit = 5 kg/m^2^)	1.01 (0.98-1.05)	**1.21 (1.01-1.44)**
Blood group O	0.75 (0.55-1.04)	0.75 (0.53-1.06)
Vicenza BS	1.01 (0.96-1.06)	1.03 (0.98-1.09)

Statistically significant associations are highlighted in bold.

BDUC, bleeding disorder of unknown cause; BMI, body mass index; BS, Bleeding Score; CFD, coagulation factor deficiency; ID, iron deficiency; MBD, mild to moderate bleeding disorder; OR, odds ratio; PFD, platelet function disorder; VWD, von Willebrand disease.

### 3.5 Association of bleeding symptoms with ID in patients with MBD

As the bleeding score was not associated with ID in univariate and multivariable logistic regression, we next investigated whether specific bleeding symptoms were associated with the occurrence of ID (Table 4). There was a trend toward higher rates of ID in patients with easy bruising than those without (41% and 34%; *P* = .06), and in female patients with postpartum hemorrhage than those without (43% and 34%; *P* = .08). Heavy menstrual bleeding was diagnosed in women with a score of ≥1 in the “menorrhagia” domain of the Vicenza bleeding score, which includes all patients with self-reported menorrhagia and those who received medical treatment (eg, hormonal therapy or surgery) for their menorrhagia prior to study inclusion, and was reported by 353 female patients (66%) and associated with ID in 155 cases (44%). However, the rate of ID was just as high in 74 out of 183 female patients without heavy menstrual bleeding (40%; *P* = .44). Surprisingly, ID occurred at a significantly lower rate in patients who had undergone surgery and had postsurgical bleeding than those without postsurgical bleeding (32% and 46%, respectively; *P* < .001). This may be explained by a higher proportion of men among patients with a history of postsurgical bleeding complications (20% vs 12%) and a higher median age among patients with postsurgical bleeding complications (45 years vs 36 years). In a multivariable regression adjusting for age, sex, blood group O, and body mass index, this statistically significant difference did not prevail (multivariable OR, 0.73; 95% CI, 0.51-1.05).

**Table 4:** Associations of bleeding symptoms with iron deficiency in patients with mild to moderate bleeding disorders.

Symptom		*n* (%)	ID, *n* (%)	*P* value^e^
HMB^a^	HMB	353 (66)	155 (44)	.44
	No HMB	183 (34)	74 (40)	
PPH^b^	PPH	122 (37)	53 (43)	.08
	No PPH	210 (63)	71 (34)	
Hematomas/easy bruising	Hematomas	442 (69)	182 (41)	.06
	No hematomas	203 (31)	68 (34)	
Intraarticular bleeding	Intraarticular bleeding	22 (3)	9 (41)	.826
	No intraarticular bleeding	622 (97)	240 (39)	
Prolonged wound bleeding	Prolonged wound bleeding	241 (37)	95 (39)	.828
	No prolonged wound bleeding	402 (63)	155 (39)	
Bleeding after TE^c^	Post-TE bleeding	214 (38)	84 (39)	.78
	No post-TE bleeding	344 (62)	131 (38)	
Epistaxis	Epistaxis	227 (35)	87 (38)	.886
	No epistaxis	419 (65)	163 (39)	
Oral/mucosal bleeding	Oral bleeding	187 (29)	71 (38)	.84
	No oral bleeding	456 (71)	177 (39)	
GI bleeding	GI bleeding	87 (13)	31 (36)	.52
	No GI bleeding	558 (87)	219 (39)	
Postsurgical bleeding^c^	Postsurgical bleeding	348 (59)	111 (32)	**<.001**^d^
	No postsurgical bleeding	239 (41)	111 (46)	
Intramuscular bleeding	Intramuscular bleeding	16 (2)	5 (31)	.54
	No intramuscular bleeding	627 (98)	243 (39)	

GI, gastrointestinal; HMB, heavy menstrual bleeding; ID, iron deficiency; PPB, postpartum bleeding; PPH, postpartum hemorrhage; TE, tooth extraction.

a Only including female patients with mild to moderate bleeding disorders.

b Only including female patients with mild to moderate bleeding disorders who delivered a child (excluding cesarean section) prior to study inclusion.

c Only including patients who had undergone surgery or TE prior to study inclusion.

d Significance prevailed after Bonferroni–Holm correction for multiple testing.

e Chi-squared test.

### 3.6 Prevalence of ID and IDA among female patients with MBD

As female sex was associated with the occurrence of ID, we next performed a separate analysis of female patients with MBD and BDUC. ID occurred at a significantly higher rate in 232 out of 540 female patients with MBD (43%) than 18 out of 106 male patients with MBD (17%; *P* < .001). However, there was no difference between female patients and 94 female healthy controls, of whom 36 had ID (38%; *P* = .398).

In female patients with MBD, the occurrence of ID was significantly higher among premenopausal women (defined as age < 55 years [23]) than postmenopausal women, as 205 of 428 premenopausal patients with MBD (48%), compared with 27 out of 112 postmenopausal patients (24%), were iron-deficient (*P* < .001). In a separate analysis of premenopausal women, however, rates of ID were similar in patients with MBD compared with 72 premenopausal healthy controls, of whom 29 (40%) had ID (*P* = .23).

For IDA, there was no significant difference between female and male patients overall, as 37 out of 540 female patients with MBD (7%) and 3 out of 106 male patients (3%) had IDA (*P* = .116). The prevalence of IDA in female patients was similar in 31 out of 428 (7%) premenopausal and 6 out of 112 (5%) postmenopausal women (*P* = .48). Similarly, the rate of IDA in premenopausal patients was similar to that of premenopausal healthy controls (6/72; 8%; *P* = .74).

## 4 Discussion

In this study, the occurrence of ID and IDA was investigated in a large cohort of patients with MBD and compared with age- and sex-matched healthy controls, which showed an overall high but similar prevalence of ID in 39% of patients with MBD and 31% of healthy controls, while the rate of IDA was low but similar in 6% of patients and 5% healthy controls. Younger age and female sex were identified as risk factors for ID in patients with MBD, while the bleeding phenotype was not associated with ID. Patients with von Willebrand disease had the highest rate of ID among patients with MBD.

Comparative data on the incidence of ID in patients with MBD are scarce and mainly focus on female patients with heavy menstrual bleeding, as ID and IDA are commonly found in those with heavy menstrual bleeding with or without bleeding disorders [2]. In line with this, we identified young age and female sex as risk factors for ID in our cohort of patients with MBD. This association has been reported in patients with bleeding disorders as well as in healthy populations [16,24]. While the rate of ID in the general population has been reported to increase rapidly around menarche, rates of ID decrease again after menopause, resulting in lower rates of ID in older populations, cumulating in an overall higher ID prevalence in individuals of menstrual age [25]. This association of younger age and ID is also apparent in our MBD patient cohort, as, in line with healthy controls, premenopausal women had significantly higher rates of ID in comparison with postmenopausal women. This increased incidence of ID in younger female patients has generally been attributed to increased blood loss from menstrual bleeding, as well as increased iron requirements during the childbearing years. In contrast, no statistically significant difference in the prevalence of IDA between male and female patients was found. This may be due to a higher rate of iron supplementation in female patients prior to study inclusion, which was recorded in almost 30% of those with heavy menstrual bleeding. Alternatively, it may be due to the successful hormonal or surgical treatment of heavy menstrual bleeding, which may have mitigated the risk for IDA in female patients [26,27].

In general, we did not find an association between ID and the presence of a bleeding disorder, such as von Willebrand disease, platelet function defects, or BDUC. This is consistent with a previous study of adolescents with heavy menstrual bleeding by Zia et al. [17], who found no difference in the incidence of ID between patients diagnosed with MBD and those with normal hemostatic assessment results. In our study of patients with MBD, the lowest prevalence of ID was found in 28% of patients with coagulation factor deficiencies, most likely due to the low proportion of women (33%) in this diagnostic subgroup. ID was diagnosed more frequently in patients with BDUC than those with platelet function defects, although without statistical significance. However, in female patients, the presence of a platelet function defect was significantly associated with a lower rate of ID in multivariable regression analysis. This is surprising considering that iron plays a critical role in platelet production and function, resulting in altered platelet morphology and impaired aggregation response in iron-deficient states. For example, Elstrott et al. [28] observed that ID reduced platelet reactivity, possibly due to decreased activity of iron-dependent enzymes involved in thromboxane synthesis. Thus, ID itself may be a significant factor contributing to platelet dysfunction and possibly MBD. However, the highest rate of ID was observed in von Willebrand disease patients, as nearly 1 in 2 patients with von Willebrand disease was iron-deficient at study inclusion. The prevalence of ID in von Willebrand disease were even higher than previously reported, as almost 50% of our von Willebrand disease patients had ID compared with a reported prevalence of ID in 22% of female patients with von Willebrand disease (29/129), which was similar to a reference cohort of nonpregnant adult women (22%) [29]. Importantly, this study likely underreported the prevalence of ID, as a more conservative ferritin cutoff of 15 ng/mL was used. Of note, in our cohort of patients with MBD and the BDUC and MBD subgroups, the vast majority of patients were premenopausal women.

As mentioned above, ID is mainly associated with heavy menstrual bleeding in women with or without bleeding disorders. In our cohort, a total of 66% of female patients reported heavy menstrual bleeding, of whom 44% had ID. The prevalence of heavy menstrual bleeding was highest in von Willebrand disease in our study, reported by 76% of female patients (data not shown). These rates of heavy menstrual bleeding are consistent with a previous study of women with low von Willebrand factor levels from the “Low von Willebrand in Ireland Cohort” Study [30]. Although heavy menstrual bleeding was reported to be even more prevalent than in our MBD patient cohort, being found in 88% of female patients (assessed and quantified by the International Society on Thrombosis and Haemostasis Bleeding Assessment Tool), the rate of ID in patients with low von Willebrand factor was 46% (with a more conservative ferritin cutoff of 23 μg/L), comparable with our patients. These data are in contrast with the prevalence of ID and IDA in adolescents with heavy menstrual bleeding reported in a study by Zia et al. [17], who found ID with ferritin levels < 15 μg/L in 61% of patients, of whom 37% had IDA. This higher rate of ID and IDA, despite the stricter diagnostic criteria for ID, may also be due to possible pretreatment of ID prior to study inclusion in our patients. A total of 64% of female patients with heavy menstrual bleeding reported previous treatment for heavy menstrual bleeding, including iron replacement, hormonal treatment, or surgery, as recorded by the Vicenza bleeding score. This prior treatment may have reduced the rate of ID at enrollment among female patients with heavy menstrual bleeding in our cohort.

Similarly, the counterintuitively significantly lower rate of ID in patients with MBD with postoperative bleeding complications than those without postoperative bleeding found in our study may be explained by the treatment of ID prior to enrollment, a higher proportion of men, and a higher age of patients with postoperative bleeding complications. In line, there was no significant association between postsurgical bleeding and ID in a multivariable regression model adjusting for the patient characteristics age, sex, body mass index, and blood group O. Of note, patients had to be free of surgery within 6 weeks before study inclusion. Nevertheless, this result may reflect more aggressive and long-term iron replacement after significant postsurgical blood loss or potential blood transfusion during and/or after surgery.

In general, ID and IDA in patients with bleeding disorders are attributed to chronic blood loss due to bleeding. However, we did not find an association between ID and the bleeding phenotype as assessed by the Vicenza bleeding score. In general, patients with MBD have a mild to moderate bleeding phenotype with limited bleeding on a daily basis, but a high risk of bleeding during hemostatic challenges. In addition, an association between body mass index and ID was found in multivariate regression analysis. Obesity has been previously associated with ID, which may be due to chronic inflammation, genetic risk factors, and pathophysiological adjustments of tissues and blood volume in obese individuals [31,32].

We did not identify a significant difference in the incidence of ID or IDA between patients with MBD and healthy controls in our study, as one-third of our healthy control cohort was also iron-deficient. While this high number suggests a possible underdiagnosis and consequently undertreatment of ID in the observed healthy cohort, it is comparable with the prevalence of ID in French women, which was estimated to be about 32% in premenopausal women and 27% in postmenopausal women [25]. Additionally, the high proportion of pretreated female MBD patients with heavy menstrual bleeding in our cohort might have artificially lowered the prevalence of ID among the patients included in this study.

To the best of our knowledge, this study provides the first comprehensive analysis of the prevalence of ID and IDA in a large cohort of patients with MBD, including patients with BDUC. However, our study has limitations. Patients, particularly those with heavy menstrual bleeding, may have received iron supplementation prior to study enrollment. Systematic documentation of prior iron supplementation was limited to female patients with heavy menstrual bleeding. Furthermore, the timing of prior iron supplementation (eg, weeks or years before enrollment or after surgery) was not recorded. Hence, these individuals were not automatically classified as iron-deficient. Prior treatments may also have increased patient awareness of ID, potentially contributing to lower observed rates of both ID and IDA in the study cohort. Additionally, patients may have undergone treatments targeting their bleeding symptoms before study inclusion. In such cases, these interventions could have mitigated symptoms and consequently lowered the risk of ID at the time of enrollment. Although data on the use of contraceptives are available, the indication of their use was not recorded. Together, these limitations may have led to an underestimation of ID and IDA among patients, especially in female patients with heavy menstrual bleeding. Lastly, men were underrepresented in the cohort of patients and healthy controls. While age- and sex-matching of patients and controls was deemed necessary, it limits the applicability of our data to the general population.

In this study, an overall high rate of ID in patients with MBD, but also in healthy controls, was shown. Among the patients with MBD, the incidence of ID was highest in patients with von Willebrand disease. Furthermore, we identified female sex, younger age, and higher body mass index as patient characteristics associated with ID in patients with MBD, but we did not find evidence for an association between ID and bleeding phenotype and severity. Furthermore, our study highlights the importance of thorough iron-status assessments in both patients and healthy people, irrespective of the presence of an inherited bleeding tendency, individual bleeding severity, and bleeding symptoms.
