# Supplementary material for: Prevalence of iron deficiency in patients with mild to moderate bleeding disorders and bleeding disorder of unknown cause
Source: Res Pract Thromb Haemost. 2025 Aug 11;9(6):102999. doi: 10.1016/j.rpth.2025.102999 (PMC12454893; doi:10.1016/j.rpth.2025.102999)
Supplement: Supplementary Material 1 [file mmc1.docx]

Prevalence of iron deficiency in patients with mild to moderate bleeding disorders and bleeding disorder of unknown cause

- Supplementary data

T. Dreier^1^, D. Mehic^1^, J. Oosterlee^1^, J. Rast^1^, A. Kaider^2^, H. Haslacher^3^, C. Ay^1^,
I. Pabinger^1^, J. Gebhart^1^

*^1^ Medical University of Vienna, Department of Medicine I, Clinical Division of Hematology and Hemostaseology, Vienna, Austria
^2^ Medical University of Vienna, Center for Medical Data Science, Institute of Clinical Biometrics, Vienna, Austria
^3^ Department of Laboratory Medicine, Vienna, Austria*

**Supplementary Table 1**: In- and exclusion criteria of the Vienna bleeding biobank

| **Inclusion criteria** | **Exclusion criteria** |
| --- | --- |
| - Age ≥ 16 years - Referred to the Hemostaseology outpatient clinic - Clinically relevant bleeding tendency - No previous diagnosis of a bleeding disorder | - Surgery or delivery within the last six weeks - Bacterial infection within the last two weeks - Current acute phase reaction - Active malignancy - Pregnancy - Thrombocytopenia (<100 x10^9^/L) - Intake of anticoagulants/antiplatelet/anti- inflammatory drugs (prior 10 days) - Continuous anticoagulant and/or antiplatelet therapy - Impaired liver function (prothrombin time <75 % of normal due to deficiency in vitamin K-dependent clotting factors) - Impaired kidney function (GFR <60 mL/min/1.73 m^2^) |

**Supplementary Table 2**: Tests performed at study inclusion

| **Global coagulation and coagulation factor tests** | |
| --- | --- |
|  | Activated partial thromboplastin time (aPTT), [seconds]  Prothrombin time (PT), [%]  Fibrinogen-Clauss [mg/dL]  Von Willebrand factor antigen (VWF:Ag), [%]  Von Willebrand factor ristocetin cofactor activity (VWF:RCo), [%]  Von Willebrand Factor Activity Glycoprotein Ib Binding Assas (VWF:GPIbM, since 2015), [%]  Factor V activity in patients with PT < 75% and aPTT > 41 seconds  Factor VII activity in patients with PT < 75%  Factor VIII activity [%]  Factor IX activity [%]  Factor XI activity in patients with aPTT > 41 seconds  Factor XIII activity [%] |
| **Platelet function – Light transmission aggregometry (LTA)** | |
|  | ADP (5μM), %  Arachidonic acid (1,6mM), [%]  Collagen (10μg/ml), [%]  Epinephrin (5,5μM), [%]  Ristocetin (1,2mg/ml), [%] |
| **Platelet function – Mepacrine release assay** | |
|  | Platelet mepacrine uptake [%] at release [%] |

Abbreviations:

ADP – adenosine diphosphate. EPI – epinephrine.

**Supplementary Table 3**: Characteristics of patients without iron status data, partial iron data and patients with complete iron status included in the study

| **Parameter** | **Patients with complete iron status assessment** | **Patients with incomplete iron status assessment** | **Patients with no iron status assessment** | **p-value** |
| --- | --- | --- | --- | --- |
| Cohort size [n, %] | 646 (69%) | 141 (15%) | 151 (16%) | n.a. |
| Female sex | 84% | 85% | 82% | 0.696*^X^* |
| Age [years]  Median, IQR | 40 [29-53] | 35 [27-49] | 37 [28-51] | 0.271*^K^* |
| Blood group O | 48% | 48% | 45% | 0.838*^X^* |
| Iron deficiency | 39% | 35% | n.a. | 0.419*^X^* |
| Bleeding score  Median, IQR | 5 (4-8) | 5 (3-8) | 6 (3-8) | 0.573*^K^* |
| Heavy menstrual bleeding reported by | 66% | 62% | 64% | 0.655*^X^* |

Abbreviations:

IQR – interquartile range.

K – Kruskal-Wallis test

X – Chi-squared test

**Supplementary paragraph 1:** Assessment of bleeding severity

Upon inclusion into the Vienna Bleeding Biobank, the clinical bleeding phenotype was evaluated by medical professionals or trained clinical personnel using the standardized Vicenza bleeding score (Vicenza-BS). The Vicenza-BS evaluates 11 symptoms (epistaxis, easy bruising/hematomas, prolonged bleeding from minor wounds, oral/mucosal bleeding, gastrointestinal bleeding, bleeding post tooth extraction, bleeding post-surgery, heavy menstrual bleeding, post-partal bleeding, intramuscular bleeding and intraarticular bleeding) for their life-long presence and severity, scoring each symptom from 0 (not present) to 3 (emergency intervention, such as blood transfusion or surgery, necessary). Hence, the Vicenza-BS can range from 0 (no pathologic bleeding symptoms) to 33 (emergency treatment for all symptoms necessary). The cut-offs for pathologic bleeding were previously defined as a total Vicenza-BS score ≥ 3 for men and ≥ 5 for women of all ages. In our study, we used both the Vicenza-BS score and the number of individual bleeding manifestations reported (out of all 11).

**Supplementary paragraph 2:** Blood sampling and storage

Blood sampling was conducted by antebrachial/antecubital venipuncture using a 21G butterfly needle set (“SAFETY Blood Collection Set”, Greiner Bio-One, Kremsmuenster, Austria) and samples were sent to the Department of Laboratory Medicine Medical University of Vienna, Vienna, Austria, within 1 hour for analysis. For blood chemistry, liver and kidney function parameters, fat metabolism and iron status, an 8 mL Serum Vacuette tube including a clotting agent and separator (Greiner Bio-One, Kremsmuenster, Austria) was used. For hemostatic tests, 3.5ml Vacuettes with sodium citrate (Greiner Bio-One, Kremsmuenster, Austria) were used. Blood cell counts, including hemoglobin and platelets, were analyzed using one 3ml EDTA Vacuette tube (Greiner Bio-One, Kremsmuenster, Austria). Platelet function tests were conducted by the Department of Transfusion Medicine and Cell Therapy, Medical University of Vienna, Vienna, Austria, using 3.5ml Vacuette tubes with sodium citrate (Greiner Bio-One, Kremsmuenster, Austria). Additionally, serum, EDTA whole-blood and citrated plasma samples were sent to the Biobank at the University Hospital Vienna, Department of Laboratory Medicine. Here, sample processing and storage is conducted according to ISO 9001:2008.

**Supplementary Figure 1**: Diagnostic workflow in die Vienna bleeding biobank


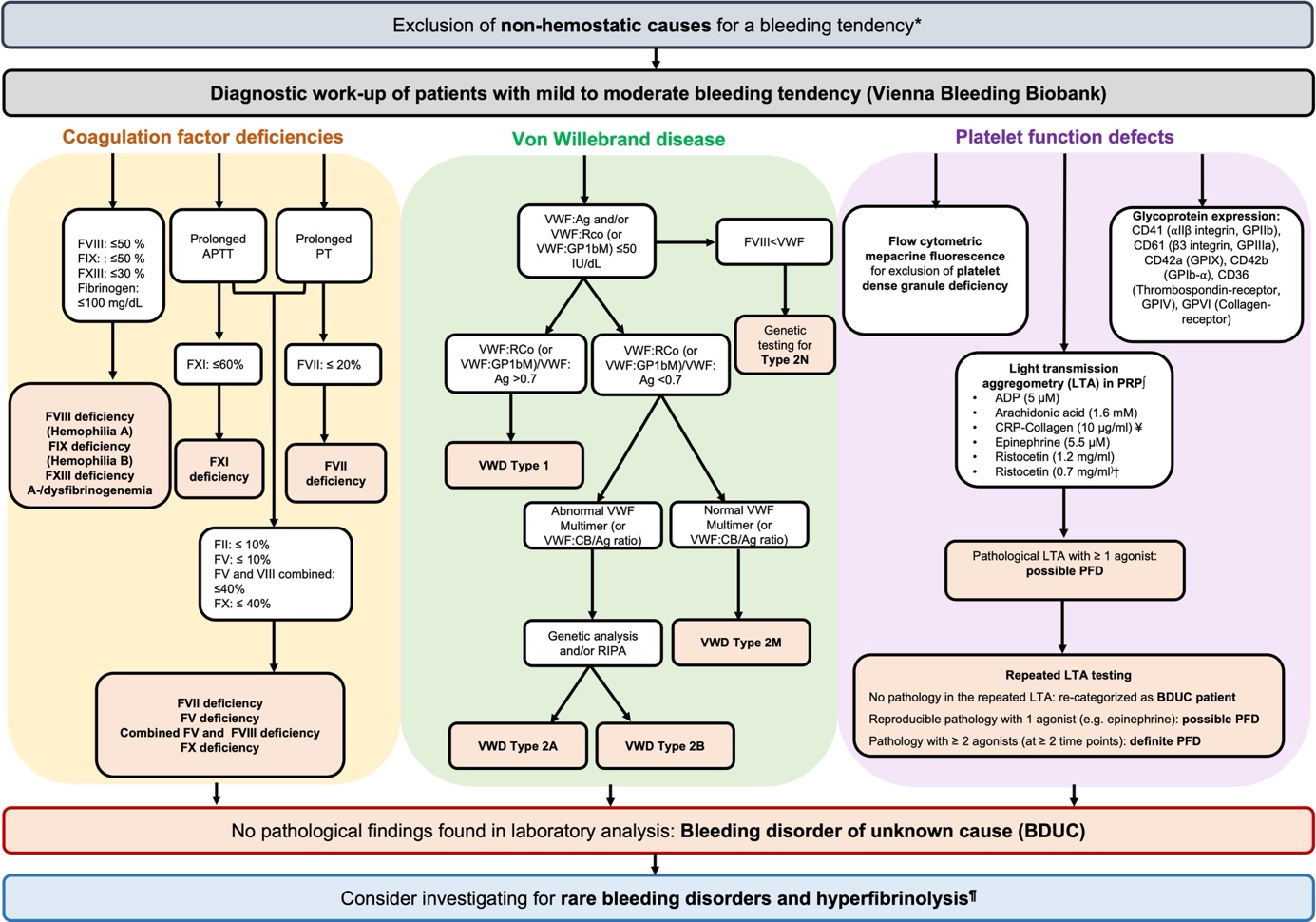


ADP – adenosine diphosphate. APTT – activated partial thromboplastin time. CD – cluster of differentiation. FV – factor V activity. FVIII – factor VIII activity. FVIX – factor IX activity. FX – factor X activity. FXI – factor XI activity- FXIII – factor XIII activity. GP – glycoprotein. PFD – platelet function defect. PT – prothrombin time. VWD – von Willebrand disease- VWF:Ag – von Willebrand factor antigen. VWF:Act – von Willebrand factor activity.

∫ Citrated blood was centrifuged at 150×g for 10 minutes at room temperature to produce platelet-rich plasma (PRP).

¥CRP-XL (0,04 μg/mL, generous gift from Dr. R. W. Farndale, Department of Biochemistry, University of Cambridge, Cambridge, UK)

† RIPA at 0.7mg/ml to identify potential PT-VWD

¶ e.g. euglobulin clot lysis time, levels of fibrinolytic factors (e.g. PAI-1, alpha2-antiplasmin, tissue plasminogen activator)

*including but not limited to Achenbach’s syndrome, Amyloidosis, Angina bullosa hemorrhagica, Auto-erythrocyte syndrome (also known as psychogenic purpura and Gardner-Diamon-Syndrome), Exercise induced purpura, Hereditary hemorrhagic telangiectasia, Hypothyroidism, Medications, Noonan syndrome, Osteogenesis imperfecta, Scurvy, Senile purpura, Skin fragility and connective tissue disorders (e.g. Ehlers-Danlos syndrome), Uremia, Vasculitis (e.g. Henoch-Schönlein purpura)

**Supplementary Figure 2**: Ferritin and transferrin saturation levels in MBD patients according to diagnosis and healthy controls


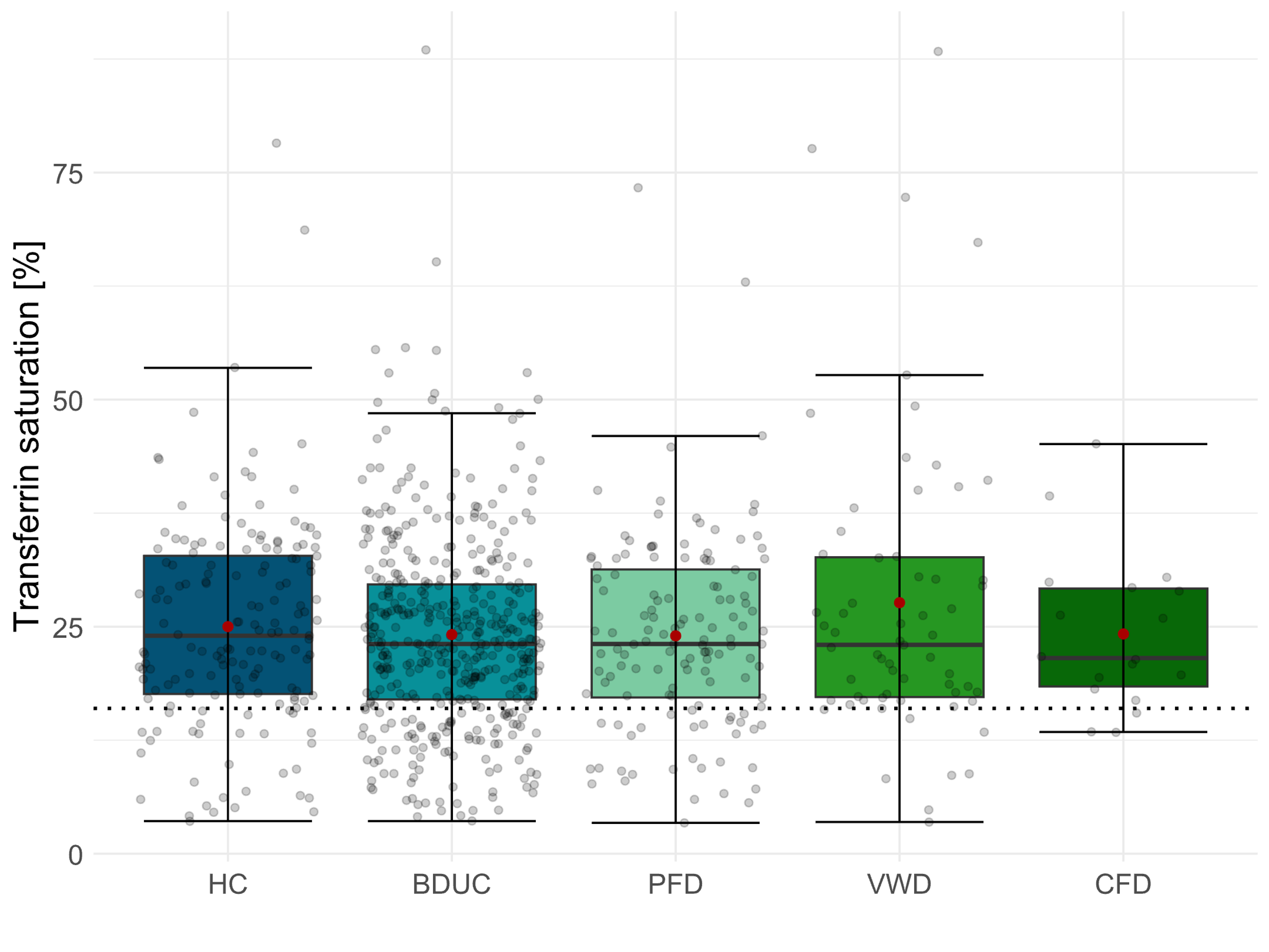

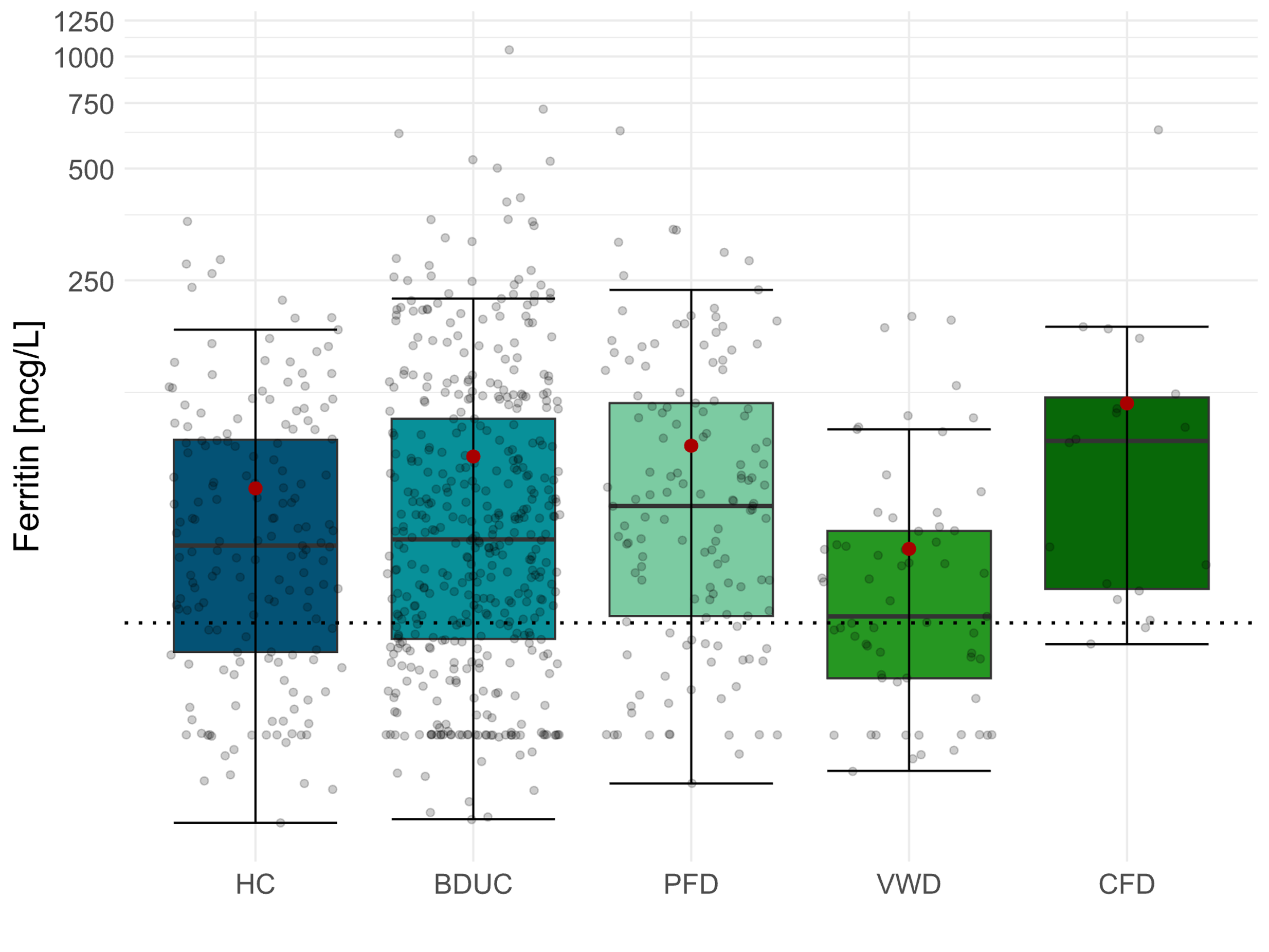


Levels of ferritin and transferrin saturation of MBD patients and HC shown as boxplots (median and quartiles). The red dot marks the mean value of ferritin/transferrin saturation. The black dotted line marks the diagnostic cutoff.

Abbreviations:
HC - Healthy controls. MBD – Mild to moderate bleeding disorders. BDUC – Bleeding disorder of unknown cause. PFD – Platelet function disorder. VWD – Von Willebrand Disease. CFD – Coagulation factor deficiencies.

**Supplementary Figure 3**: ID and IDA in patients and controls.

Patients with IDA

**n = 40**

Non-anemic ID patients

n = 210

Ferritin ≤ 30 or
Tf-Sat < 16%

MBD Patients included

n = 646

Patients with ID

**n = 250**

Patients without ID

n = 396

Hb < 13 for men
Hb < 12 for women

Healthy controls with IDA

**n = 6**

Non-anemic ID Healthy controls

n = 31

Ferritin ≤ 30 or
Tf-Sat < 16%

Healthy controls included

n = 118

Healthy controls with ID

**n = 37**

Healthy controls without ID

n = 81

Hb < 13 for men
Hb < 12 for women

Abbreviations:

MBD – Mild to moderate bleeding disorders. BDUC – Bleeding disorder of unknown cause. PFD – Platelet function disorder. VWD – Von Willebrand Disease. CFD – Coagulation factor deficiencies. Hb – Hemoglobin. Tf-Sat – Transferrin-saturation. ID – Iron deficiency. IDA – Iron deficiency anemia.
